# Comprehensive proteomic analysis of exosome mimetic vesicles and exosomes derived from human umbilical cord mesenchymal stem cells

**DOI:** 10.1186/s13287-022-03008-6

**Published:** 2022-07-15

**Authors:** Zhaoxia Zhang, Tao Mi, Liming Jin, Mujie Li, Chenghao Zhanghuang, Jinkui Wang, Xiaojun Tan, Hongxu Lu, Lianju Shen, Chunlan Long, Guanghui Wei, Dawei He

**Affiliations:** 1 Department of Urology, Children’s Hospital of Chongqing Medical University, Yuzhong District, Chongqing, 400014 People’s Republic of China; 2 Chongqing Key Laboratory of Children Urogenital Development and Tissue Engineering, Chongqing, 400014 People’s Republic of China; 3 China International Science and Technology Cooperation Base of Child Development and Critical, National Clinical Research Center for Child Health and Disorders, Chongqing, Ministry of Education Key Laboratory of Child Development and Disorders, Chongqing Key Laboratory of Pediatrics, Chongqing, 400014 People’s Republic of China

**Keywords:** Mesenchymal stem cells, Exosomes, Exosome mimetic vesicles, Proteomics

## Abstract

**Background:**

Exosomes derived from mesenchymal stem cells (MSCs) have shown to have effective application prospects in the medical field, but exosome yield is very low. The production of exosome mimetic vesicles (EMVs) by continuous cell extrusion leads to more EMVs than exosomes, but whether the protein compositions of MSC-derived EMVs (MSC-EMVs) and exosomes (MSC-exosomes) are substantially different remains unknown. The purpose of this study was to conduct a comprehensive proteomic analysis of MSC-EMVs and MSC-exosomes and to simply explore the effects of exosomes and EMVs on wound healing ability. This study provides a theoretical basis for the application of EMVs and exosomes.

**Methods:**

In this study, EMVs from human umbilical cord MSCs (hUC MSCs) were isolated by continuous extrusion, and exosomes were identified after hUC MSC ultracentrifugation. A proteomic analysis was performed, and 2315 proteins were identified. The effects of EMVs and exosomes on the proliferation, migration and angiogenesis of human umbilical vein endothelial cells (HUVECs) were evaluated by cell counting kit-8, scratch wound, transwell and tubule formation assays. A mouse mode was used to evaluate the effects of EMVs and exosomes on wound healing.

**Results:**

Bioinformatics analyses revealed that 1669 proteins in both hUC MSC-EMVs and hUC MSC-exosomes play roles in retrograde vesicle-mediated transport and vesicle budding from the membrane. The 382 proteins unique to exosomes participate in extracellular matrix organization and extracellular structural organization, and the 264 proteins unique to EMVs target the cell membrane. EMVs and exosomes can promote wound healing and angiogenesis in mice and promote the proliferation, migration and angiogenesis of HUVECs.

**Conclusions:**

This study presents a comprehensive proteomic analysis of hUC MSC-derived exosomes and EMVs generated by different methods. The tissue repair function of EMVs and exosomes was herein verified by wound healing experiments, and these results reveal their potential applications in different fields based on analyses of their shared and unique proteins.

**Supplementary Information:**

The online version contains supplementary material available at 10.1186/s13287-022-03008-6.

## Background

Mesenchymal stem cells (MSCs) were first discovered in the 1960s [1]. Among the most important adult stem cells, MSCs are derived from the mesoderm and have the ability to differentiate into osteoblasts, chondrocytes, or adipocytes [2, 3]. MSCs are widely used in the field of regenerative medicine because they have angiogenic, antiapoptotic, and immune regulation abilities, among other functions [4]. Clinical trials of MSC-related therapies have been registered in the world's largest clinical trial database, ClinicalTrial, and a total of 1082 trials with MSCs have been carried out worldwide. Among these studies, 252 clinical trials, the most in the world, were conducted in China, followed by 200 trials in the United States (http://www.clinicaltrials.gov/, accessed on August 2020). MSCs are derived from a wide range of source tissues, such as bone marrow, umbilical cord, adipose, liver, and synovial tissues [5]. Among these MSCs, umbilical cord MSCs (UC MSCs) are considered to be preferred for drug testing in the fields of regenerative medicine and cell therapy because of the following advantages: fast self-renewal ability, strong doubling ability, stable doubling time, low immunogenicity, and lack of ethical issues [6]. Initial research suggested that MSCs are best used in regenerative medicine on the basis of the "transplantation differentiation hypothesis," which emphasizes that MSCs can differentiate into a variety of different types of cells due to their high differentiation potential, enabling them to replace cells lost at injury sites or in dead tissues [4, 7]. However, although an increasing number of recent studies have shown that MSC-based treatment shows significant efficacy in various disease models, the currently available data are insufficient to confirm that exogenous cells successfully replace cells in damaged tissue because few cells that are transplanted survive [8, 9]. These observations indicate that the tissue-repairing effect of MSCs is not due to cell transplantation and differentiation. Other studies showed that conditioned medium (CM) obtained from MSC culture promoted bone healing in mice with calvarial defects and exerted therapeutic effects in lung injury models [10, 11]. Therefore, some researchers have proposed that MSCs repair these tissues through paracrine functions and not through cell replacement [12, 13]. Genomic studies have confirmed that MSCs secrete biologically active proteins [14, 15]. Therefore, cell-free therapy has attracted increasing attention, and researchers hope to find substances with similar components that have the same effects as cells successfully used in replacement cell therapy.

Exosomes are spherical particles that are released outside the cell after the fusion of multivesicular bodies with the cell membrane [16]. Exosomes have a lipid bilayer membrane structure composed of diglycerides, phospholipids, glycerophospholipids, polyglycerophospholipids, and high levels of cholesterol and sphingolipids [17, 18]. Compared with plasma membranes, exosomal membranes are more rigid and more stable in the external environment. Exosomes have diameters of approximately 40–150 nm. The sizes of exosomes prepared through different methods slightly differ. Exosomes prepared for transmission electron microscopy (TEM) analysis are usually smaller in diameter than those prepared for nanoparticle tracking analysis (NTA) due to sample dehydration [19]. Exosomes carry a variety of genetic materials, including microRNAs (miRNAs), mRNAs, and proteins, which are similar to those in the parent cells. Studies have demonstrated that exosomes can regulate the activities of target cells through the proteins that they transport to target cells [20, 21]. Exosomes secreted by MSCs have been proven to play therapeutic roles in a variety of injury types and diseases, including acute and chronic kidney injury, spinal cord injury, myocardial ischemia, skin wounds, and peripheral nerve injury [22–27]. MSC-derived exosomes (MSC-exosomes) exert no harmful effects on the liver or kidney, and exosomes do not cause tumors or tumor spreading, in contrast to MSCs [28]. Based on these findings, MSC-exosomes are expected to be widely used in new cell-free therapies for various diseases, including those currently treated with regenerative medicines. However, the low level of exosomes released from cells greatly hinders their clinical application. Therefore, we are eager to find a method to increase the yield of exosomes that are similar in structure, composition and function to MSC-exosomes.

Exosome mimetic vesicles (EMVs) are produced by the continuous extrusion of cells in a mini-extruder with different sized polycarbonate membrane filters [29]. EMVs are similar to exosomes in structure, size, and components, including various RNAs and proteins, and can transfer the substances that they carry from parent cells to target cells [30]. The yield of EMVs is several-fold higher than that of exosomes, and their expression levels of RNAs and proteins are also several-fold higher than those in exosomes [31]. Most research has focused on the function of EMVs as drug delivery vehicles. EMVs derived from stem cells have been confirmed to play roles in promoting angiogenesis and neuroprotection [32]. EMVs derived from MSCs can reduce inflammation in endothelial cells and repair spinal cord injury [33, 34]. In summary, studies have shown the substantial promise of MSC-derived EMVs (MSC-EMVs) in the field of regenerative medicine, and they are therefore worthy of in-depth study.

Proteins have important cellular functions, and an increasing number of research groups have focused their attention on exosomal proteins. To date, more than 1000 proteins have been identified in MSC-exosomes [35]. Exosomal proteins have been proven to be involved in many biological processes, including biogenesis, cell communication, development and tissue repair and regeneration [36], indicating the importance of proteins for MSC-exosome functions. MSC-EMVs, as exosome analogs, have not been investigated through proteomic analysis. Therefore, we performed proteomic analyses of MSC-EMVs and MSC-exosomes. We then compared the two sets of data to observe differences, performed data analyses of MSC-EMV, and MSC-exosome components, and simply explored the effects of exosomes and EMVs on wound healing ability to provide a new theoretical basis for EMV applications.

## Materials and methods

### Cell culture

MSCs obtained from human umbilical cord (hUC MSCs) were obtained at the Stem Cell Center of the Children’s Hospital of Chongqing Medical University. To generate exosomes, cells were maintained in DMEM/F12 supplemented with 10% exosome-free fetal bovine serum (FBS). Exosomes in FBS were removed by ultracentrifugation overnight at 100,000 × *g* and then filtered through a 0.22-µm filter (Millipore). To generate EMVs, cells were maintained in complete DMEM/F12 supplemented with 10% FBS and incubated at 37 °C with 5% CO_2_. Human umbilical vein endothelial cells (HUVECs) were purchased from the Cell Bank of the Chinese Academy of Sciences (Shanghai, China). The cells were cultured in high-glucose DMEM supplemented with 10% fetal bovine serum and 1% penicillin streptomycin solution (Gibco, USA) at 37 °C and 5% CO_2_.

### Exosome isolation

Exosomes were isolated from CM through differential centrifugation. The medium was collected from hUC MSCs when they reached 90% confluence. In summary, the CM was first subjected to serial centrifugation to remove cells (300 × *g*, 10 min) and cellular debris (2000 × *g*, 20 min). The CM was centrifuged at 10,000 × *g* for 30 min to remove large microvesicles. Later, the supernatant was subjected to centrifugation at 100,000 × *g* for 70 min at 4 °C to pellet the exosomes. The exosome pellets were resuspended in phosphate-buffered saline (PBS, Invitrogen) and centrifuged again at 100,000 × *g* for 70 min at 4 °C. Finally, the exosome pellets were resuspended in PBS and filtered (through 0.22-μm filters) to remove large particles.

### EMV generation

EMVs were extracted following a previously reported protocol [29]. hUC MSCs were harvested, resuspended in PBS at a concentration of 1 × 10^6^/ml and extruded in a mini-extruder with polycarbonate membrane filters with various pore sizes (10, 5, and 1 µm) (Avanti Polar Lipids). The extruded samples were collected and subjected to ultracentrifugation at 100,000 × *g* for 1 h at 4 °C. After ultracentrifugation, the precipitates were resuspended in PBS and then filtered (with 0.22-µm filters) to ultimately obtain EMVs.

### Transmission electron microscopy

Transmission electron microscopy (TEM) was used to confirm the presence of exosomes and EMVs. Approximately, 20 µl of exosomes and EMVs were added separately to copper grids. All excess fluids were removed using filter paper, and the samples were negatively stained with 2% uranyl acetate for 30 s. The grids were rinsed in deionized water and allowed to dry overnight. The samples were then air-dried using an electric incandescent lamp and viewed using an electron microscope (Hitachi, S-3000N).

### Nanoparticle tracking analysis

The exosome and EMV particles were resuspended in PBS, and their sizes and concentrations were analyzed by NTA. Then, exosomes and EMVs diluted in solution were injected into the LM10 unit (Malvern Panalytical). NTA software, version 2.3 (Malvern Panalytical, England), was used to collect and analyze the videos.

### Western blot analysis

Total proteins were isolated from exosomes and EMVs using RIPA lysis buffer (Beyotime, China) with phenylmethanesulfonyl fluoride (PMSF; Beyotime, China) and centrifuged at 12,000 × *g* for 20 min at 4 °C. The concentrations were measured using a bicinchoninic acid (BCA) assay. Then, 10 μg of total protein was added to polyacrylamide gels, separated by sodium dodecyl sulfate–polyacrylamide gel electrophoresis (SDS–PAGE), and transferred onto polyvinylidene difluoride membranes. The membranes were blocked with 5% nonfat milk for 1 h and then incubated with different monoclonal primary antibodies overnight at 4℃. Primary antibodies against CD63 (1:500, Abcam, USA), Alix (1:1000, Abcam, USA), and TSG101 (1:1000, Abcam, USA) were used. After washing in Tris-buffered saline/Tween (TBST), the membranes were incubated with goat anti-rabbit or mouse antibodies (1:5000, Zhongshan, China) for 1 h at 37 °C. The immunoblots were visualized using Immobilon Western Chemiluminescent HRP Substrate (Millipore, USA).

### Liquid chromatography with tandem mass spectrometry (LC–MS/MS) analysis

Each sample was separated using an Easy nLC system at a nanoliter flow rate. The chromatographic column was equilibrated with 100% solution A (0.1% formic acid in water), and the sample was loaded onto an analytical column (Thermo Fisher Scientific, Acclaim PepMap RSLC 50 µm × 15 cm, nano viper, P/N164943) via an autosampler for separation. The flow rate was 300 nL/min. Solution B was comprised of 0.1% formic acid in acetonitrile aqueous solution; after chromatographic separation, the sample was analyzed with a Q Exactive plus mass spectrometer. The analysis time was 60–90 min, detection was performed in positive ion mode, the scanning range of the precursor ion was 350–1800 m/z, and the primary mass spectrum resolution was 70,000. Then, Proteome Discoverer 2.1 (Thermo Fisher Scientific) software was used to convert the original map file (.raw file) generated with Q Exactive Plus software into a.mgf file, which was submitted to the MASCOT2.6 server for database retrieval. Then, the database search file (.dat file) obtained from the MASCOT server was transmitted back through Proteome Discoverer 2.1 software. The data were filtered on the basis of a false discovery rate (FDR) < 0.01 to obtain highly reliable qualitative results.

### Bioinformatics analyse*s*

#### Differentially expressed proteins

The exosome-available data from the ExoCarta database (http://www.exocarta.org) was used to compare the identified proteins. Protein differential expression analysis between two different groups was performed with R software (R4.0.1, the EDGER package). Differences in protein expression with a *p* value < 0.05 and an absolute fold change ≥ 2 were considered to denote differentially expressed proteins.

#### Gene Ontology (GO) enrichment analysis

Blast2GO was used to perform GO annotations of target protein sets. Proteins were classified into biological process, cellular compartment and molecular function GO categories. For data in each category, a two-tailed Fisher’s exact test was performed to determine the enrichment of differentially expressed proteins compared to all identified proteins. GO terms with a corrected *p* value < 0.05 were considered to indicate significant enrichment.

#### Kyoto Encyclopedia of Genes and Genomes (KEGG) enrichment analysis

KEGG Orthology (KO) and Links Annotation (KOALA) software was used to analyze the KEGG GENES database to classify target protein sequences identified by KO analysis, and through this process, information on pathways in which the target protein sequence is involved, as indicated by KO classification, was automatically obtained. Fisher’s exact test was performed to compare the KEGG pathways involving target proteins and the overall protein sets to evaluate the significance level of protein enrichment in specific KEGG pathways. The KEGG analysis results with a corrected *p* value < 0.05 were considered to indicate significant enrichment.

#### Gene set enrichment analysis

Gene set enrichment analysis (GSEA) was performed using the gene set c2.cp.v7.2.symbols.gmt. Pathway. Significantly enriched protein sets were defined as protein sets with a *p* value < 0.05, a normalized enrichment score (NES) > 1 or < − 1, and an FDR < 0.25.

#### Protein–protein interaction network analysis

The STRING database (https://string-db.org/) was used to screen different groups of protein–protein interaction (PPI) networks with an interaction score ≥ 0.4. The Cytoscape cytoHubba plug-in was used to find and identify the 20 most connected hub proteins in each network.

#### Screening of differentially expressed membrane proteins

First, the proteins located in the cell membrane were selected based on their localization. Second, membrane proteins with high expression in exosomes and EMVs (top 4) were screened according to the expression level detected by sequencing.

### Wound healing experiments in vitro and in vivo

#### Labeling and tracing of exosomes and EMVs in HUVECs

The EMVs and exosomes were labeled with the membrane dye PKH26 in accordance with the manufacturer's instructions. HUVECs were seeded in 24-well plates containing cell sheets, and PKH26-labeled EMVs and exosomes were then added at a concentration of 100 µg/ml. After culturing for 6 h, 12 h and 24 h, the cells were washed with PBS, fixed with 4% paraformaldehyde, stained with DAPI, and photographed under a fluorescence microscope (Nikon, K10587, Japan).

#### Migration assay

Six-well plates were used for the scratch wound assay. HUVECs were seeded in plates at 200,000 cells/well and incubated until reaching confluence, after which a scratch was made using a pipet tip. The exfoliated cells were washed with PBS and cultured in serum-free medium with or without exosomes or EMVs (100 µg/ml). Images were acquired at 0 h, 12 h and 24 h, and the cell migration distance was measured using NIS-Elements (Nikon, Japan) analysis software. The migration distance (%) was calculated using the following formula: (initial wound width—final wound width)/ initial wound width × 100.

Transwell assays were performed using 24-well transwell plates (Corning, Corning, NY, USA) with 8-µm-pore size filters. HUVECs were routinely digested and resuspended in serum-free medium, and 10,000 cells were seeded into the upper chamber. Medium containing 10% fetal bovine serum was added to the lower chamber, which either contained or did not contain EMVs or exosomes (100 µg/ml). The cells were routinely cultured for 24 h, fixed with 4% paraformaldehyde, stained with crystal violet, photographed under a microscope, and counted by ImageJ software.

#### Proliferation assay

Cell Counting Kit-8 analysis was performed to assess cell proliferation. When the HUVECs reached a confluence of approximately 80%, 2000 cells/well were inoculated into 96-well plates. The medium was supplemented with or without EMVs and exosomes (100 µg/ml) derived from MSCs. The cells were cultured routinely, and 10 µl of CCK-8 solution and fresh medium were added to each well at 0 h, 24 h, 48 h, and 72 h. The cells were incubated for another 2 h, and the absorbance was detected at 450 nm using a microplate reader.

#### Tube formation assay

The matrix gel without growth factors was pre-added to 96-well plates (50 µl/well) and incubated at 37 °C for 30 min to allow solidification. A total of 20,000 cells/well were seeded in plates containing the matrix gel, and the medium was supplemented with or without EMVs or exosomes (100 µg/ml). After culturing for 6 h, tube formation was observed under a microscope. Image-J software was used to measure the number of the branches.

#### Animals and treatment

Adult male BALB/C mice with an average weight of 20–25 g were purchased from the Experimental Animal Center of Chongqing Medical University (SCXK 2018-0003). All mice were reared at the Animal Center, Children's Hospital of Chongqing Medical University (SYXK 2017-0012) and provided adequate water and food. Under isoflurane (2.5%) anesthesia, the hair on the back of the mouse was shaved, and a full-thickness skin wound (about 10 mm in diameter) was created on the back. Eighteen mice were randomly divided into three groups. The mice were subcutaneously injected with an equal amount of PBS (100 µl), EMVs or exosomes (200 µg dissolved in 100 µl of PBS) at multiple points around the wound for 7 days. Skin incision healing was observed and photographed on days 0, 2, 4 and 7, and the incision area was measured by ImageJ. Wound closure (%) = (the initial wound area – At)/ the initial wound area × 100, where At is the wound area at day 2, 4 or 7 post-operation. The underside of the skin was observed and photographed on day 7 after wounding to examine the formation of new blood vessels. All experiments were approved by the Animal Research Committee of Children's Hospital of Chongqing Medical University.

### Statistical analysis

Principal component analysis (PCA) was performed to evaluate quantitative protein repeatability. Data are presented as the mean ± standard deviation (SD). One-way analysis of variance (ANOVA) was used to compare the means of multiple groups, and the means between two groups were compared using an independent-sample t test. Graph Pad Prism 8.0 (GraphPad Software, San Diego, CA, USA) was used for all statistical analyses. *P* values < 0.05 were considered to indicate statistical significance. All experiments were performed three times.

## Results

### Extraction of EMVs and exosomes

**Fig. 1:**
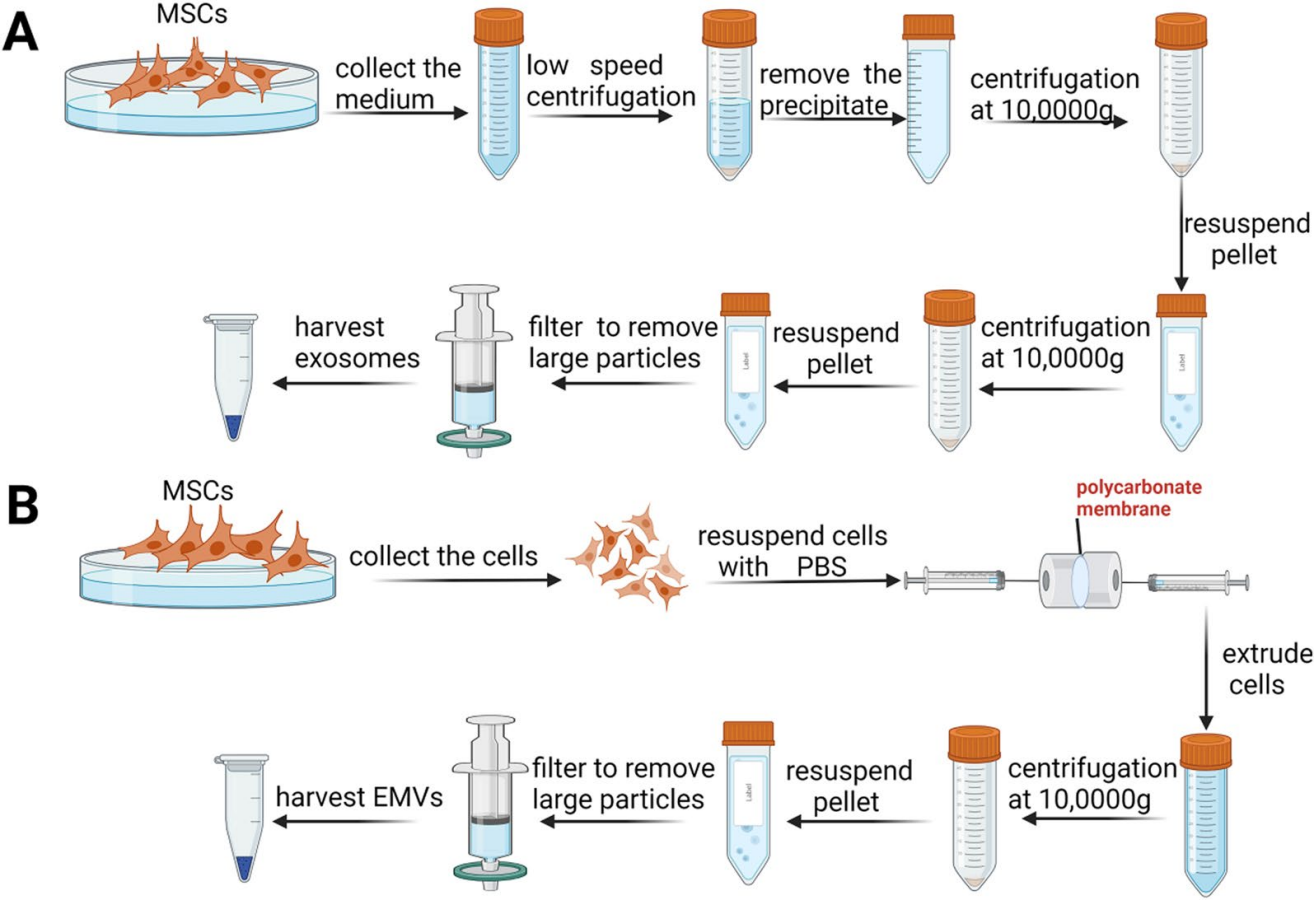
Schematic diagram of the generation of hUC MSC-exosomes and hUC MSC-EMVs. Flow chart for isolating MSC-exosomes (**A**). Flow chart for generating MSC-EMVs (**B**)

EMVs were extracted from MSCs, and exosomes were obtained from the CM supernatant of MSC cultures (Fig. 1). To obtain exosomes, CM from cell culture was collected and subjected to differential ultracentrifugation (Fig. 1A). For harvesting EMVs, cells were resuspended in PBS and extruded multiple times through polycarbonate membranes with various pore diameters (10 μm, 5 μm, and 1 μm) to generate crude EMVs (Fig. 1B). After these extrusions were performed, the crude EMVs were ultracentrifuged at 100,000 × *g* for 1 h at 4 °C, and the pellet was resuspended in PBS and then passed through a 0.22-μm filter to remove debris and obtain purified EMVs.

### Characterization of MSC-EMVs and MSC-exosomes

**Fig. 2:**
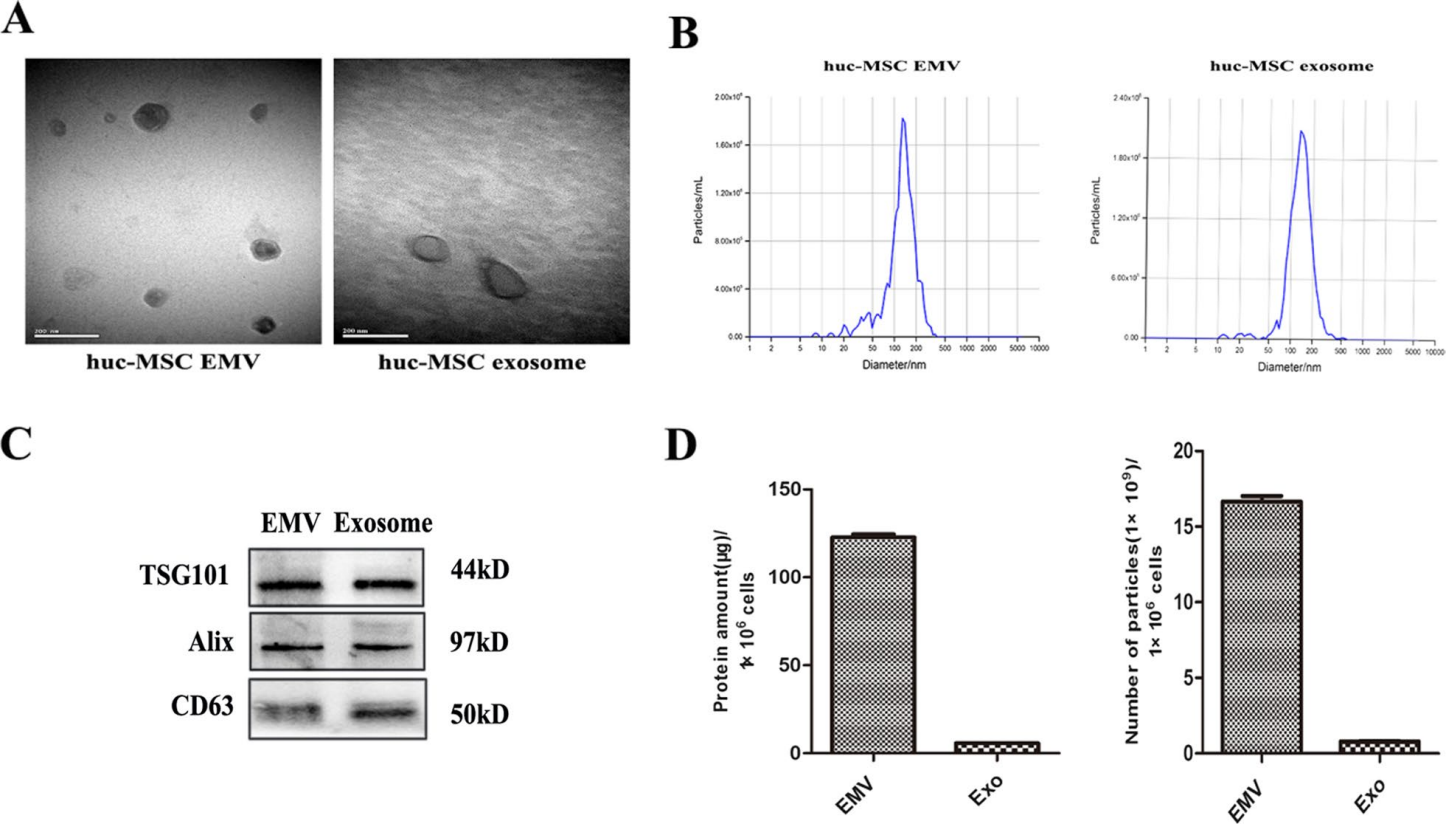
Characterization of hUC MSC- EMVs and hUC MSC-exosomes. TEM images of MSC-EMVs and MSC-exosomes. Scale bar: 200 nm (**A**). Size distribution of MSC-EMVs and MSC-exosomes as measured by NTA (**B**). The expression levels of TSG101, Alix and CD63 in MSC-EMVs (10 μg) and MSC-exosomes (10 μg) as detected via western blot analysis (**C**). The yields of EMVs and exosomes measured as the total proteins and particle numbers (*n* = 3) (**D**). Exo: exosomes

EMV and exosome morphology was examined by TEM. MSC-EMVs and MSC-exosomes were enclosed within a lipid membrane and formed rounded cup-shaped vesicles ranging from 30 to 100 nm in diameter (Fig. 2A). NTA verified that the EMVs and exosomes had mean diameters of 126.9 ± 3.0 nm and 133.0 ± 2.4 nm, respectively (Fig. 2B). The differences in the size distributions of EMVs and exosomes measured by NTA and TEM may have been due to sample dehydration [19]. In order to verify that both EMVs and exosomes can express exosomal markers such as TSG101, Alix, and CD63, different doses of EMVs and exosomal proteins were used for WB analysis (rendered in original WB image in Fig. 2C), and in the main text, only the expression of exosomal markers in the same dose of EMVs (10 ug) and exosomes (10 ug) were showed (Fig. 2C). The protein level in MSC-EMVs (122.8 µg) was more than 20-fold (average) higher that in MSC-exosomes (5.8 µg) obtained from the same number of MSCs (1 × 10^6^ cells). The number of MSC-EMV particles (approximately 16 × 10^9^ particles) was also more than 20-fold higher than that of exosome particles (nearly 0.8 × 10^9^ particles) obtained from the same number of MSCs (Fig. 2D).

### Proteomic analysis of MSC-EMVs and MSC-exosomes

Most of the peptides contained 7–20 amino acids, which conformed to the general rules of trypsin enzymatic hydrolysis and higher-energy C-trap dissociation (HCD) fragmentation. The distribution of peptide lengths as identified by MS met the quality control requirements (Additional file 1: Fig. S1A). Most proteins corresponded to more than two peptides (Additional file 1: Fig. S1B). The molecular weight of most protein pairs ranged from 10 to 100 kDa (Additional file 1: Fig. S1C). The coverage of the protein was positively correlated with its abundance in the sample (Additional file 1: Fig. S1D). The PCA diagram indicated good repeatability (Additional file 1: Fig. S1E).

**Fig. 3:**
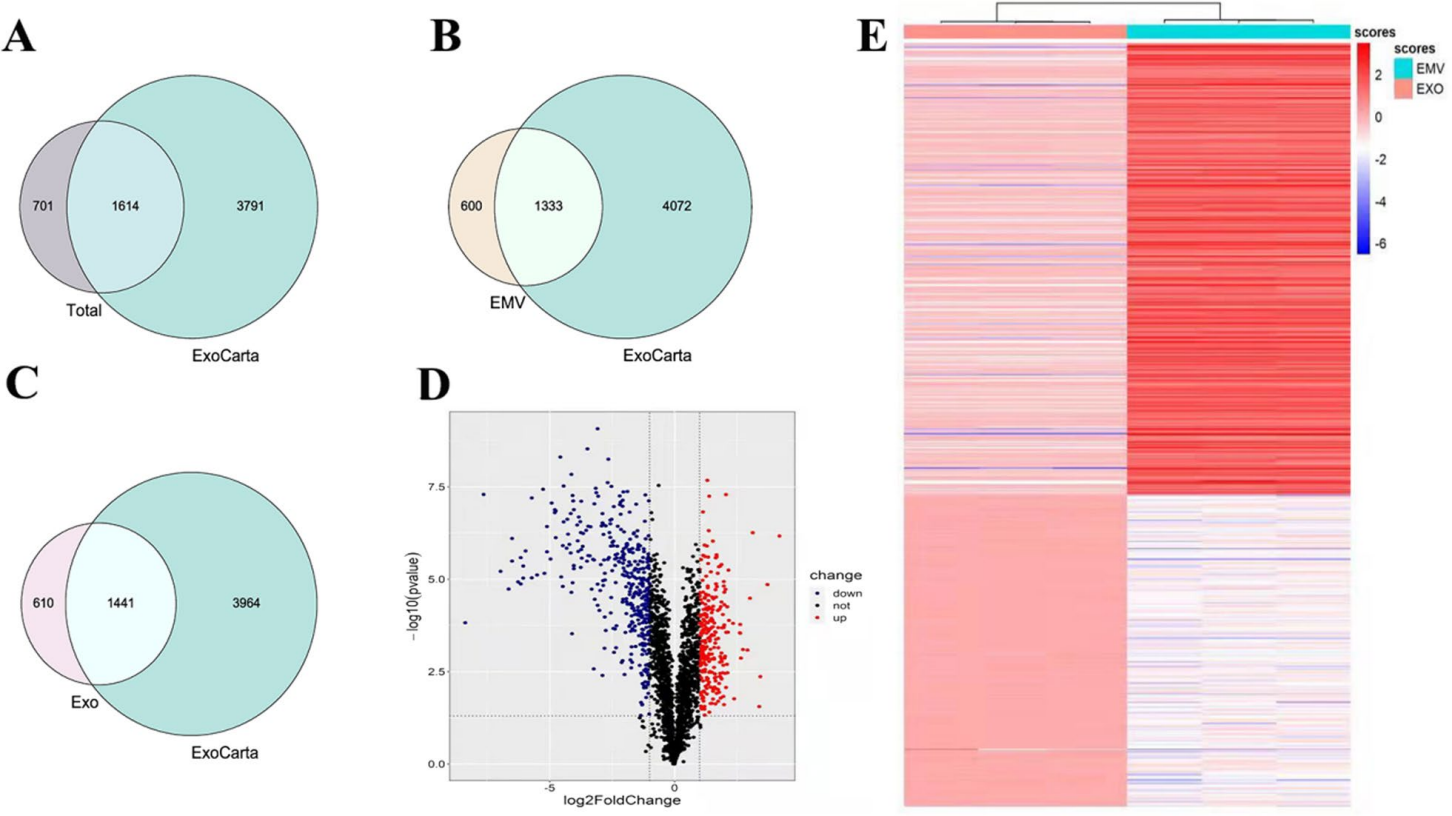
Proteomic analysis of hUC MSC-EMVs and hUC MSC-exosomes. Venn diagram of the quantified total proteins (Total) against ExoCarta (**A**). Venn diagram of MSC-EMVs (EMVs) against ExoCarta (**B**). Venn diagram of MSC-exosomes (Exos) against ExoCarta (**C**). Volcanogram of differential proteins for MSC-EMVs versus MSC-exosomes (**D**). Heatmap of the protein levels of the differentially expressed proteins (**E**)

Proteomic analyses of the MSC-EMVs and MSC-exosomes revealed 2315 proteins in the two groups. The quantified total proteins were compared with ExoCarta (an exosome database), and most of the proteins were found in the ExoCarta database (Fig. 3A). There were 1933 and 2051 proteins in the MSC-exosomes and MSC-EMVs, respectively. The proteins in the two groups were compared with ExoCarta, and most of them were found in the ExoCarta database (Fig. 3B, C). The volcano map shows a total of 646 differentially expressed proteins between the two groups (Fig. 3D), of which 264 were highly expressed in EMVs and 382 were enriched in exosomes. Subsequently, the differential proteins were hierarchically clustered. These proteins are presented in an expression heatmap (Fig. 3E).

### Bioanalysis of proteins common to EMVs and exosomes

**Fig. 4:**
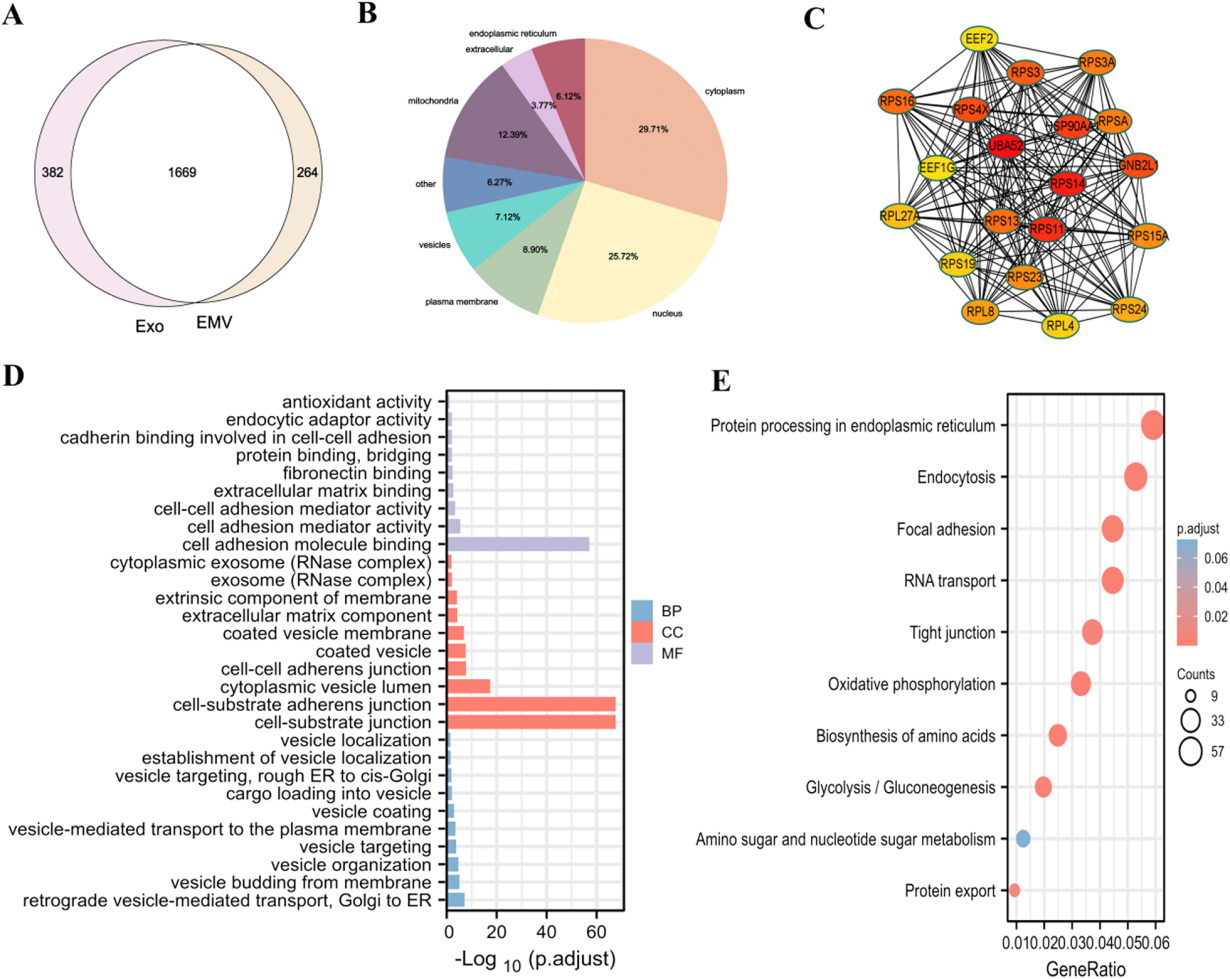
Bioanalysis of proteins common to hUC MSC-EMVs and hUC MSC-exosomes. Venn diagrams of proteins in hUC MSC-EMVs and hUC MSC-exosomes (**A**). The cytolocalization of the shared proteins between hUC MSC-EMVs and hUC MSC-exosomes (**B**). PPI network of the shared proteins (**C**). GO analysis of the shared proteins (**D**). KEGG analysis of the shared proteins (**E**)

Using a Venn diagram to compare the proteins in the two groups, 1669 proteins were found to be in both groups, 382 proteins were unique to exosomes, and 264 proteins were unique to EMVs (Fig. 4A). A localization analysis of the shared proteins was performed, and the results showed that the shared proteins were mainly located in the cytoplasm, followed by the nucleus (Fig. 4B). GO analysis of the shared proteins showed that the proteins in the cellular component category were mainly concentrated in cell-substrate junctions and cell-substrate adherens junctions; in the biological process category, these proteins were enriched in retrograde vesicle-mediated transport and vesicle budding from the membrane; and in the molecular function category, most of the shared proteins were enriched in cell adhesion molecule binding and cell adhesion mediator activity (Fig. 4D). KEGG analysis of the shared proteins showed that they were enriched in protein processing in the endoplasmic reticulum (Fig. 4E). A PPI network analysis of the shared proteins (20 proteins with the strongest interactions were selected for mapping) identified the key proteins as ubiquitin A-52 (UBA52), ribosomal protein S11 (RPS11) and ribosomal protein S14 (RPS14) (Fig. 4C).

### Bioanalysis of specific proteins in MSC-exosomes

**Fig. 5:**
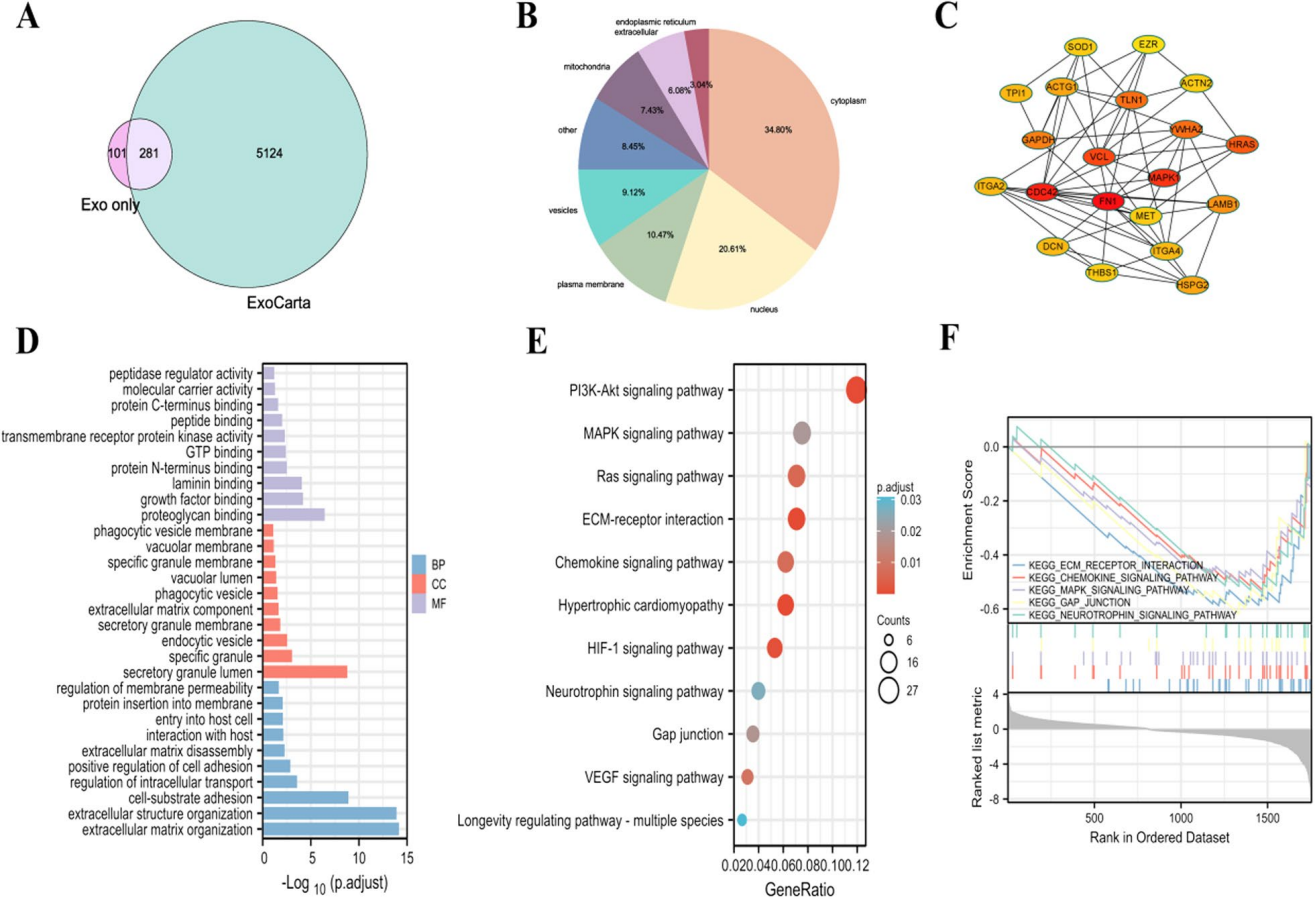
Bioanalysis of specific proteins in hUC MSC-exosomes. Venn diagrams of specific proteins in hUC MSC-exosomes (Exo only) against ExoCarta (**A**). Cytolocalization of specific proteins in hUC MSC-exosomes (**B**). PPI network of specific proteins in hUC MSC- exosomes (**C**). GO analysis of specific proteins in hUC MSC-exosomes (**D**). KEGG analysis of specific proteins in hUC MSC-exosomes (**E**). GSEA of exosome-specific proteins (**F**)

Proteins unique to exosomes were compared with those in the ExoCarta database, and 74% of the MSC-exosomal proteins were found in the database (Fig. 5A). Localization analysis of exosome-specific proteins was conducted, and the results showed that exosome-specific proteins were mainly located in the cytoplasm and nucleus, followed by cellular membranes and vesicles (Fig. 5B). GO analysis of the specific proteins in exosomes showed that most of the proteins in the cell component category were concentrated in the secretory granule lumen, specific granules and endocytic vesicles; in the biological process category, these proteins were enriched in extracellular matrix organization and extracellular structural organization; and in the molecular function category, the proteins were enriched in proteoglycan binding and growth factor binding (Fig. 5D). KEGG analysis revealed that the proteins were enriched in the PI3K-AKT, MAPK, and RAS signaling pathways and the ECM-receptor interaction pathway (Fig. 5E). The GSEA results showed that exosome-specific proteins were enriched in the ECM-receptor interaction pathway (NES of − 1.83), gap junction pathway (NES of − 1.64), chemokine signaling pathway (NES of − 1.60), MAPK pathway (NES of − 1.57), and neurotrophin signaling pathway (NES of − 1.55) (Fig. 5F). PPI analysis of proteins specific to exosomes identified fibronectin-1 (FN1), cell division cycle 42 (CDC42) and mitogen-activated protein kinase 1 (MAPK1) as the key proteins (Fig. 5C).

### Bioanalysis of specific proteins in MSC-EMVs

**Fig. 6:**
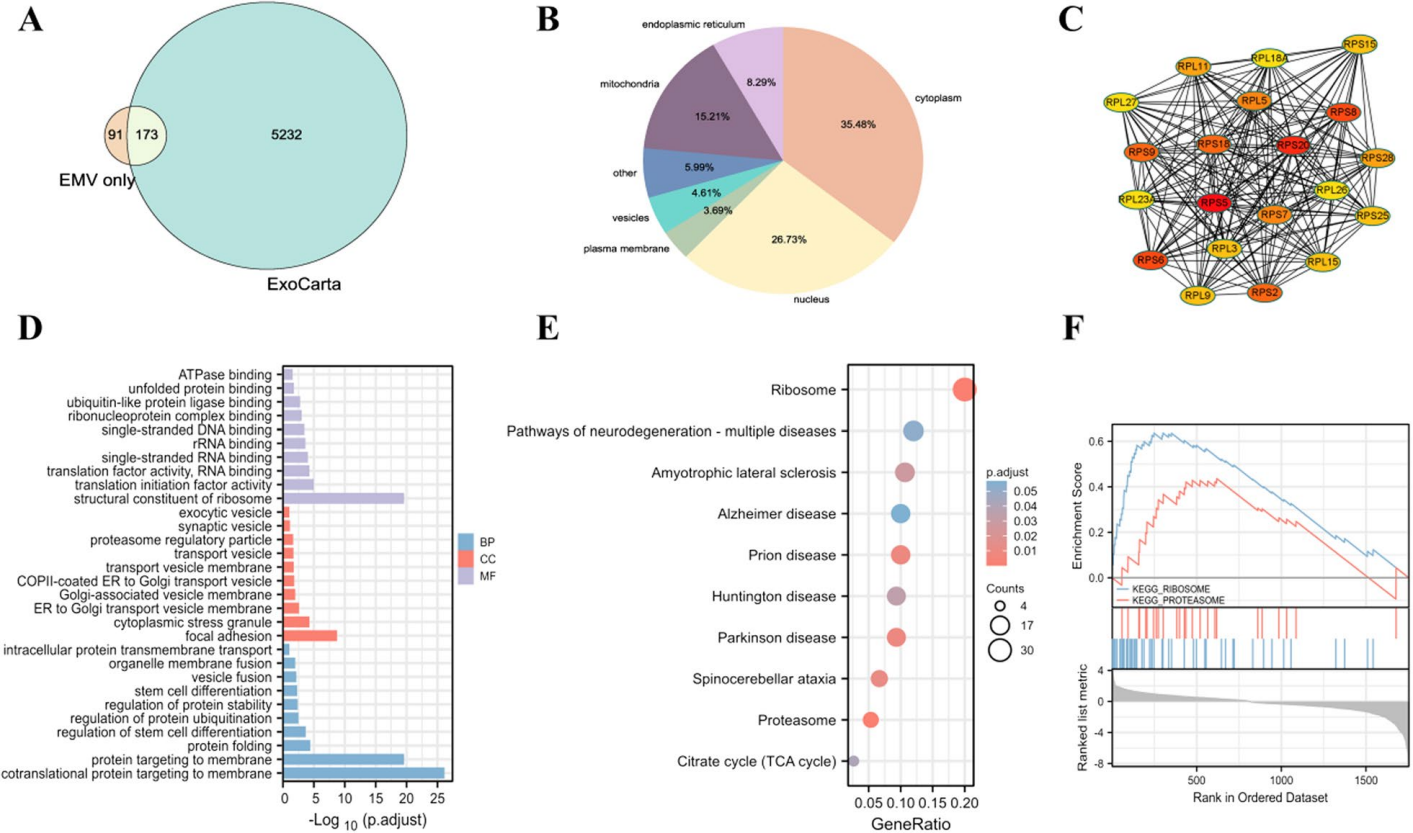
Bioanalysis of specific proteins in hUC MSC-EMVs. Venn diagrams of specific proteins in hUC MSC-EMVs (EMV only) against ExoCarta (**A**). Cytolocalization of specific proteins in hUC MSC-EMVs (**B**). PPI network of specific proteins in hUC MSC-EMVs (**C**). GO analysis of specific proteins in hUC MSC-EMVs (**D**). KEGG analysis of specific proteins in hUC MSC-EMVs (**E**). GSEA of exosome-specific proteins (**F**)

The specific proteins in EMVs were compared with those in the ExoCarta database, and 66% of the MSC-EMV proteins matched proteins in the database (Fig. 6A). The localization of the EMV-specific proteins was analyzed, and the results showed that the EMV-specific proteins were mainly located in the cytoplasm and nucleus (Fig. 6B). GO analysis of the EMV-specific proteins showed that most proteins in the cell component category were concentrated in focal adhesion and cytoplasmic stress granules; proteins in the biological process category, including cotranslational proteins, mainly targeted the membrane; and the proteins in the molecular function category were mainly structural constituents of ribosomes and showed translation initiation factor activity (Fig. 6D). KEGG analysis showed that these proteins were enriched in ribosomal pathways, neurodegenerative diseases, and pathways of multiple disease pathways (Fig. 6E). The GSEA results showed that the EMV-specific proteins were enriched in the ribosome pathway with an NES of 2.86 and the proteasome pathway with an NES of 1.62 (Fig. 6F). PPI network analysis identified ribosomal protein S5 (RPS5) and ribosomal protein S5 (RPS20) as the key proteins (Fig. 6C).

### Differentially expressed membrane proteins in MSC-exosomes and MSC-EMVs

**Fig. 7:**
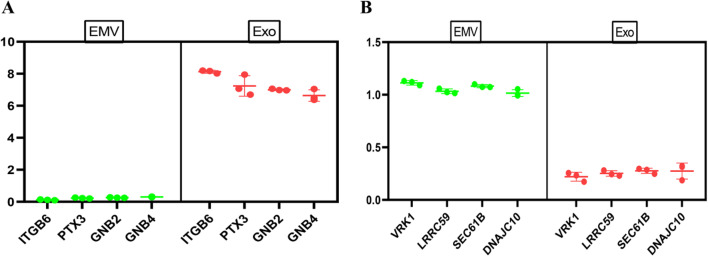
Differentially expressed membrane proteins in hUC MSC-exosomes and hUC MSC-EMVs. Membrane proteins enriched in hUC MSC-exosomes (**A**). Membrane proteins enriched in hUC MSC-EMVs (**B**). The vertical axis represents the expression levels of membrane proteins obtained by sequencing

Finally, membrane proteins differentially expressed in exosomes and EMVs were analyzed, and the results showed that integrin beta 6 (ITGB6), Pentraxin 3 (PTX3), G protein subunit beta 2 (GNB2) and G protein subunit beta 4 (GNB4) were highly expressed in exosomes (Fig. 7A), while Vaccinia-related kinase 1(VRK1), SEC61B, DnaJ heat shock protein family (Hsp40) member C10 (DNAJC10) and leucine-rich repeat-containing 59 (LRRC59) were enriched in EMVs (Fig. 7B).

### HUVECs internalize MSC-exosomes and MSC-EMVs

We examined whether HUVECs could internalize MSC-exosomes and MSC-EMVs.

**Fig. 8:**
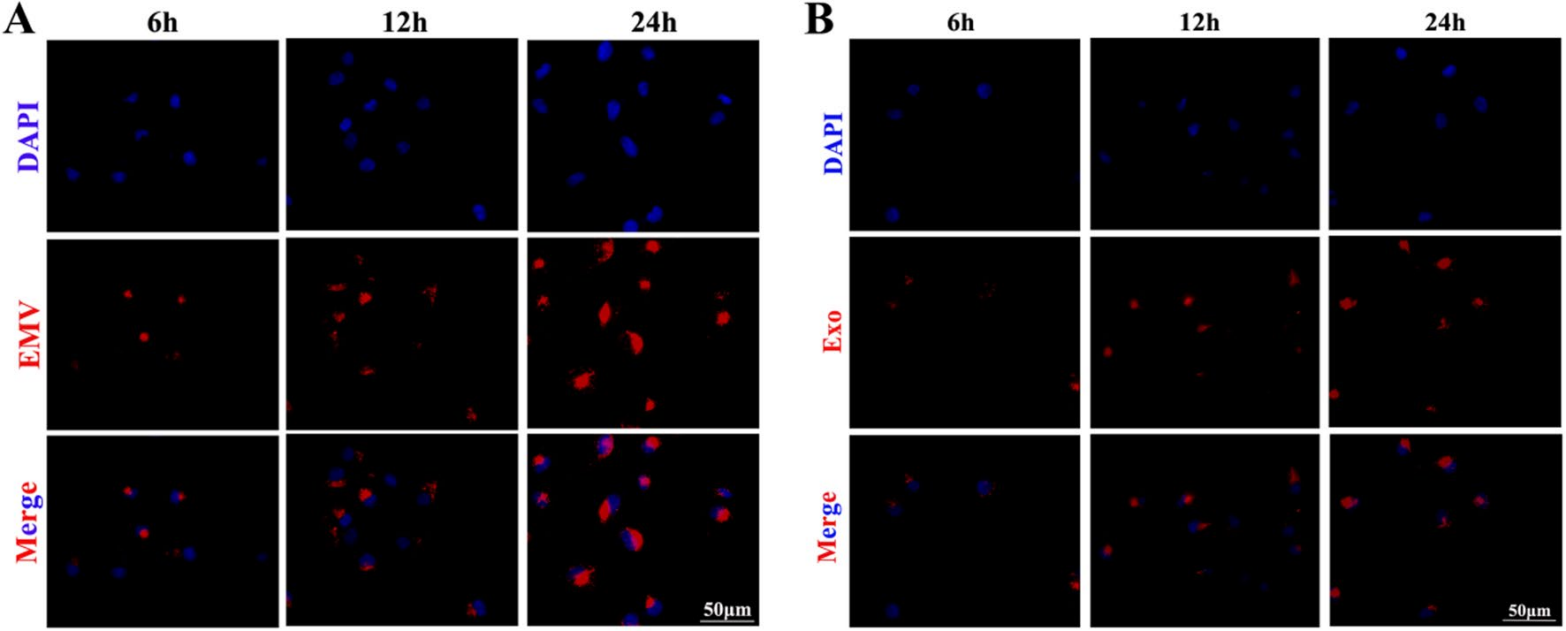
HUVECs internalize MSC-exosomes and MSC-EMVs. Fluorescence microscopy of HUVECs and MSC-EMVs (EMV) after coincubation for 6, 12, and 24 h. Scale bar: 50 µm (**A**). Fluorescence microscopy of HUVECs and MSC-exosomes (Exo) after coincubation for 6, 12, and 24 h. Scale bar: 50 µm (**B**)

The results showed that HUVECs could take up EMVs and exosomes; The EMVs and exosomes were shown to be endocytosed into the cytoplasm at 6 h and 12 h and to aggregate along the nuclear membrane at 24 h (Fig. 8A, B).

### Proangiogenic effects of EMVs and exosomes on HUVECs

**Fig. 9:**
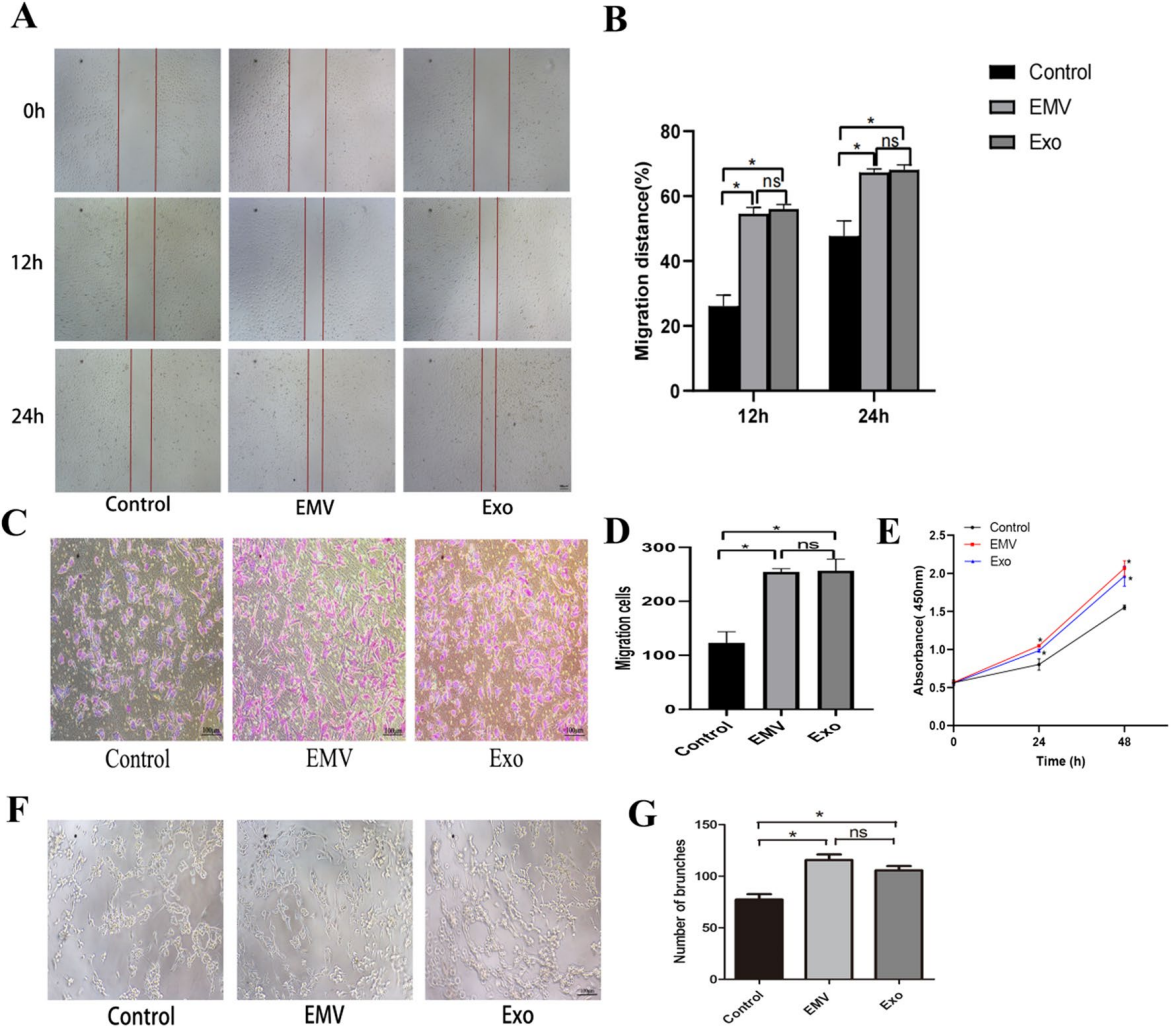
Proangiogenic effects of EMVs and exosomes on HUVECs. EMVs and exosomes (Exo) promoted the migration of HUVECs as determined by the scratch wound assay (**A**, **B**) and Transwell assay (**C**, **D**). The proliferation of cells in the different groups as determined by the CCK-8 assay (**E**). EMVs and exosomes (Exo) increased the tube formation ability of HUVECs (**F**, **G**). Scale bar: 100 μm (**A**, **C**, **F**). *P < 0.05 compared with the control group. ns, the EMV group versus the Exo group

The scratch wound (Fig. 9A, B) and transwell assay (Fig. 9C, D) results showed that the cell migration abilities of the EMV- and exosome-treated groups were significantly enhanced compared with the control group but not significantly different between themselves. CCK-8 analysis showed that the cell proliferation abilities of the EMV and exosome groups were significantly enhanced compared with the control group not significantly different between themselves (Fig. 9E). The tube formation assay results showed that the tube formation abilities of the EMV and exosome groups were significantly enhanced compared with the control group but not significantly different between themselves (Fig. 9F, G).

### Both MSC-EMVs and MSC-exosomes promote cutaneous wound healing in mice

**Fig. 10:**
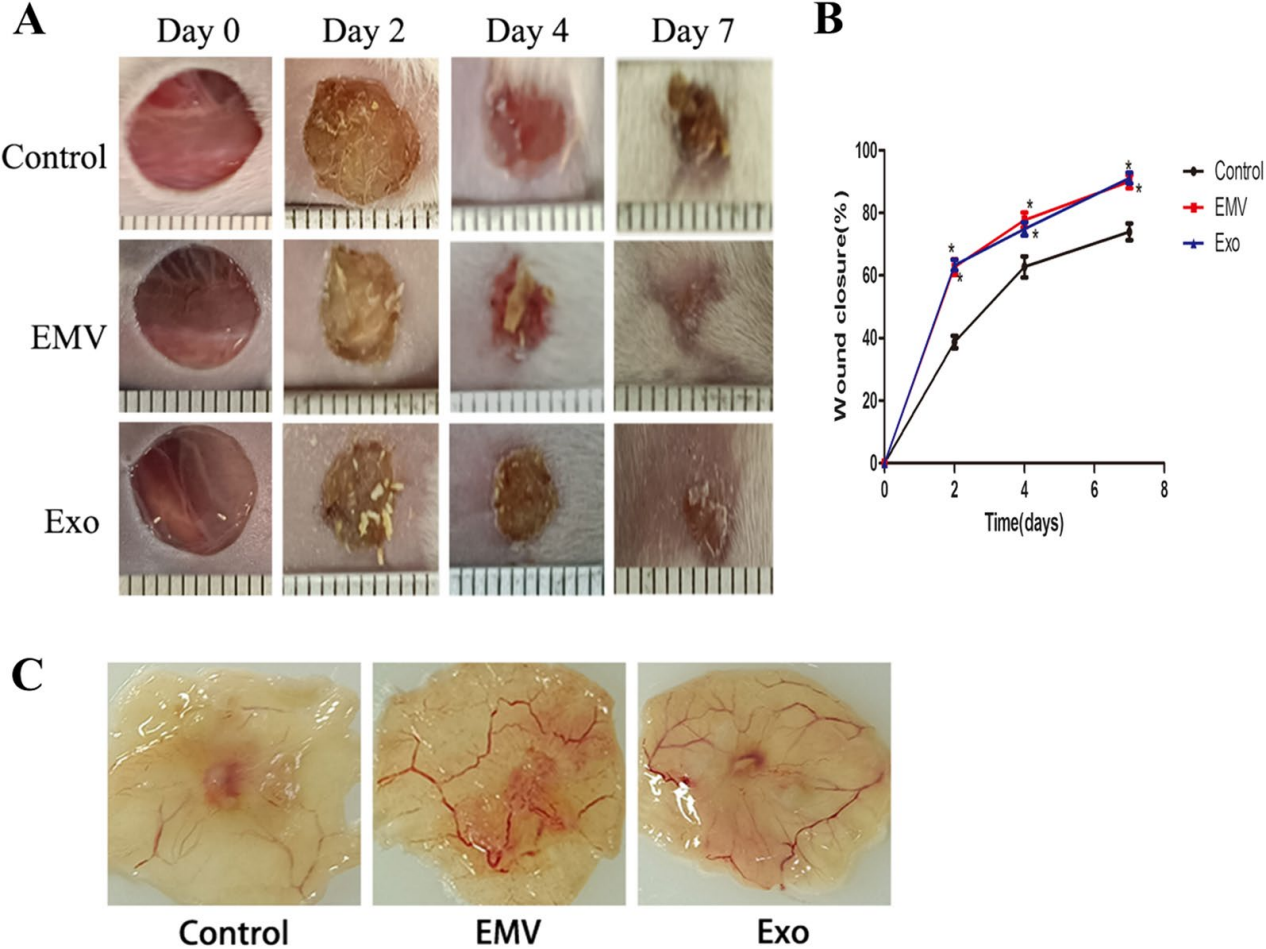
Both MSC-EMVs and MSC-exosomes promote cutaneous wound healing in mice. Gross views of wounds and the rates of wound closure in the EMV, exosome and PBS control groups on days 0, 2, 4 and 7 after wounding. *n* = 6 per group (**A**, **B**). Gross views of wounds on day 7 post wounding. Newly formed blood vessels were detected at the wound sites. *n* = 6 per group (**C**). *P < 0.05 compared with the PBS group (control)

Compared with that in the PBS group, the closure of skin wounds on the backs of mice in the EMV and exosome groups was accelerated, and the wounds in the EMV group and exosome group were smaller on 2, 4 and 7 days. Wound size did not significantly differ between the EMV and exosome groups (Fig. 10A, B). Compared with those in the PBS group, newly formed blood vessels were more numerous in wounds treated with EMVs and exosomes on day 7 after injury (Fig. 10C).

## Discussion

As a new cell-free therapy, MSC-exosomes have been widely used in the field of regenerative medicine. However, the number of exosomes secreted by cells is low; approximately, 1–4 µg of exosomes is secreted per 1 million MSCs, which makes it difficult to meet the levels needed in clinical trials and animal experiments [37]. In 2013, some scholars proposed a new method to produce EMVs from cells by continuous extrusion through polycarbonate membrane filters of different sizes in a mini-extruder. EMVs generated through this method were similar in structure and size similar to exosomes and expressed exosomal markers; however, their yield was greater than that of exosomes [29]. Initially, EMVs were mainly used to encapsulate chemotherapy drugs for tumor research. However, research advances have led to the gradual use of EMVs for tissue repair.

Proteins perform many functions, and numerous studies have focused on the proteomics of exosomes derived from MSCs [38, 39]. However, few studies have reported a proteomic analysis of EMVs. A study focused on the proteomics of EMVs and exosomes derived from neuroblastoma cells showed that EMVs and exosomes shared most proteins, but proteins unique to EMVs and exosomes were also found [40]. MSCs play important roles in the field of regenerative medicine, and the proteomes of EMVs derived from MSCs are worthy of further exploration. Therefore, our research aimed to compare and analyze the proteomes of MSC-EMVs and MSC-exosomes. We found that MSC-EMVs were similar to MSC-exosomes in structure and size and that MSC-EMVs expressed proteins that have been recognized as highly enriched exosomal markers, such as ALIX, a vesicle transport-related protein in cells,CD63 (a four-transmembrane protein), and TSG101, a protein involved in exosome biogenesis [41]. The EMV yield was superior to that of exosomes in terms of proteins and particle number, consistent with the literature [29, 42, 43].

In addition, while EMVs and exosomes expressed their own unique proteins, more than 80% of the proteins were common to both EMVs and exosomes, which was potentially due both EMVs and exosomes carrying the genetic material of their parent cells. We compared the proteins of exosomes and EMVs with those in the ExoCarta database and found that approximately 70% of the proteins in both groups were in the database, indicating that the materials we extracted were consistent with known exosome components. Previous studies showed that proteins related to the extracellular matrix and cell adhesion were highly enriched in exosomes [44]. Our study showed that the shared proteins in the two groups were also related to cell matrix and cell adherens junctions, which indicated that EMVs are associated with locations enriched with exosomes, consistent with previous studies on exosomes [45]. Protein localization analysis of exosomes and EMVs revealed that most of the proteins were localized in the cytoplasm and nucleus; more EMV-specific proteins than exosome-specific proteins were located in mitochondria and the endoplasmic reticulum, while more exosome-specific proteins than EMV-specific proteins were localized in vesicles and the plasma membrane. EMVs are directly formed through direct cell extrusion and may contain all organelle components, which indicates that their composition, functions, and protein localization may be more complicated than those of exosomes. Ignoring the complexity of their components, hUC MSC-EMVs were similar to exosomes in protein composition and function; because of their superior yield, we speculate MSC-EMVs will replace exosomes in the future.

In addition, our research showed that the key shared proteins were UBA52, RPS11 and RPS14. UBA52 was confirmed to be highly expressed in the serum exosomes of patients with metastatic gastric cancer [46]. RPS11 is a ribosomal subunit, and a reduction in RPS11 expression can inhibit protein synthesis [47]. The expression of RPS11 was confirmed to be associated with susceptibility to TOP2 poisons across multiple cancer cell lines, including glioma cells [48]. The deletion of RPS14 is closely related to del(5q) myelodysplastic syndrome (MDS) [49]. The key exosome-specific proteins were FN1, CDC42 and MAPK1. FN1 is regarded as a classic component of the extracellular matrix that can regulate cell adhesion, differentiation and other processes. FN1 has also been confirmed to be a component of liver cell-derived extracellular vesicles (EVs) that can promote EV uptake by targeting cells [50]. Upon vascularization, the cell division cyclin CDC24 enables SCAP-Exos (exosomes derived from stem cells in the apical papilla) to effectively promote craniofacial soft tissue regeneration [51]. Previously, researchers showed that MAPK1 may be closely related to renal fibrosis. The key EMV-specific proteins were determined to be RPS5 and RPS20. RPS5 is highly expressed in psoriatic arthritis and is expected to become a biomarker [52]. The pathogenic variant of RPS20 was previously associated with familial early-onset colorectal cancer [53, 54]. Our results showed that the key proteins in the two groups were related to various diseases and biological processes. Importantly, these key proteins may be related to the specific therapeutic effects of exosomes and EMVs on certain diseases.

We also compared the membrane proteins enriched in exosomes and EMVs, identifying the membrane proteins ITGB6, PTX3, GNB2, and GNB4 as being mainly enriched in exosomes. High expression of ITGB6 can promote the proliferation of bile duct epithelial cells [55], and PTX3 is a soluble pattern recognition receptor. Studies have shown that PTX3 is mainly released from cells through conventional protein secretion, but only a small portion of PTX3 is released by exosomes derived from adipocytes stimulated by lipopolysaccharide (LPS) [56]. Mutations and overexpression of GNB2 can cause leukemia, and downregulation of GNB2 expression reduces the cell proliferation potential and confers survival benefits [57]. A study identified GNB4 mutations as a cause of Charcot-Marie-Tooth disease (CMT) and emphasized the importance of Gβ4-related GPCR signals for human peripheral nerve function [58]. A previous study also showed that the membrane proteins VRK1, SEC61B, DNAJC10 and LRRC59 were mainly enriched in EMVs. VRK1 is a nuclear Ser/Thr chromatin kinase that is overexpressed in many types of tumors and associated with poor prognosis [59]. SEC61B is a subunit of the SEC61 transposon complex and is commonly considered to be a marker of the endoplasmic reticulum (ER). The absence of SEC61B leads to an increase in the fluidity of the translocon complex and a decrease in the number of membrane-bound ribosomes [60]. DNAJC10 is a member of the DNAJC protein family, a subclass of heat shock proteins; mutations of these proteins may be related to Parkinson's disease (as they are a feature of Parkinson's disease and other neurological diseases) [61]. The leucine-rich repeat sequence containing 59 (LRRC59) is strictly required for the import of exogenous fibroblast growth factor 1 (FGF1) into the nucleus, where it shows growth-regulating activity, and FGF1 can be shuttled to the cytoplasm and nucleus by endocytic vesicles [62]. Considering these studies, we suspected that these differentially expressed membrane proteins endow exosomes and EMVs with the ability to target key components in certain diseases.

In summary, the components of EMVs produced by continuous extrusion may be more complex and contain exosomes. Based on this, a comprehensive proteomic analysis of EMVs and exosomes can further our understanding of the similarities and differences in the protein components of EMVs and exosomes and provide a better theoretical basis for the treatment of different diseases. Our study also confirmed that EMVs and exosomes from hUC MSCs shared approximately 80% of the same proteins and contained unique proteins. However, which type (EMVs or exosomes) is key for the therapeutic effect remains unknown. Both exosomes and EMVs are speculated to play key roles in some diseases if the shared proteins have major therapeutic effects; if the therapeutic effect is correlated to a greater extent with the specific proteins of EMVs, EMVs may play a more critical role. Conversely, if the therapeutic effect is more related to exosome-specific proteins, exosomes may have a more critical role.

There have been many studies on MSCs in the field of regenerative medicine. For example, many basic and clinical studies have proven that the injection of hUC MSCs and their CM can promote wound healing by stimulating angiogenesis [62]. The genetic materials of EMVs and exosomes are derived from their parental cells, and EMVs and exosomes can theoretically participate in angiogenesis and wound healing processes, such as reepithelialization, neovascularization/vascular maturation and other biological processes [63]. Therefore, we conducted a preliminary analysis of the tissue repair function of EMVs and exosomes, and wound healing experiments in vivo confirmed that the local transplantation of EMVs and exosomes to wounded sites on mouse skin induced significant regeneration, mainly by accelerating the wound closure process and promoting collagen deposition. In vitro experiments confirmed that HUVECs endocytosed EMVs and exosomes, and both EMVs and exosomes promoted the proliferation, migration and tube formation of HUVECs. In vivo and in vitro experiments confirmed that EMVs and exosomes had similar effects on promoting wound healing. Wound healing is a complex process involving multiple cell types, including fibroblasts and vascular endothelial cells, and insufficient local angiogenesis is considered to be an important factor underlying poor chronic wound healing [64, 65]. Angiogenesis plays an important role in wound healing, and the formation of new blood vessels after birth mainly provides oxygen and nutrition for the wound to maintain fibroblast proliferation, collagen synthesis and reepithelialization [66]. Exosomes from various sources have been confirmed to promote wound healing by participating in angiogenesis [67–69], which is consistent with our research conclusion. We speculate that the similar effects of EMVs and exosomes on wound healing is due to their shared proteins.

## Conclusions

To our knowledge, this is the first study to report comprehensive proteomic analyses of EMVs and exosomes derived from hUC MSCs. We found that EMVs were similar to exosomes in structure, shape and size but were produced at a higher yield. We compared the shared and unique proteins in the two groups and analyzed their functions. Moreover, we preliminarily explored the roles of EMVs and exosomes in tissue repair by performing wound healing experiments. We believe that most of the shared proteins will lead to the replacement of exosomes with EMVs in future applications. However, the differentially expressed proteins specifically enriched in exosomes, and EMVs may be potential therapeutic targets in some diseases.

## Supplementary Information

**Additional file 1: Fig. S1.** Sample repeatability test and quality control analysis of the mass spectrometry data. Peptide length distribution (**A**). Number of peptides per protein distributed (**B**). Mass distribution of the identified proteins (**C**). Protein sequence coverage distribution (**D**). PCA diagram (**E**).

## Data Availability

The datasets presented in this study can be found in online respositories. Data are available via ProteomeXchange with identifier PXD033899.The names of the repository/repositories and accession number can be found below:https://www.ebi.ac.uk/pride/archive/projects/PXD033899/publish.

## Abbreviations

MSCs: Mesenchymal stem cells
MVs: Exosome mimetic vesicles
hUC MSCs: Human umbilical cord MSCs
CM: Conditioned medium
TEM: Transmission electron microscopy
NTA: Nanoparticle tracking analysis
LC–MS/MS: Liquid chromatography with tandem mass spectrometry
GO: Gene ontology
KEGG: Kyoto Encyclopedia of Genes and Genomes
GSEA: Gene set enrichment analysis
PPI: Protein–protein interaction network
