# Supplementary material for: Comprehensive proteomic analysis of exosome mimetic vesicles and exosomes derived from human umbilical cord mesenchymal stem cells
Source: Stem Cell Res Ther. 2022 Jul 15;13:312. doi: 10.1186/s13287-022-03008-6 (PMC9284776; doi:10.1186/s13287-022-03008-6)
Supplement: Supplementary file 1 — Additional file 1: Fig. S1. Sample repeatability test and quality control analysis of the mass spectrometry data. Peptide length distribution (A). Number of peptides per protein distributed (B). Mass distribution of the identified proteins (C). Protein sequence coverage distribution (D). PCA diagram (E). [file 13287_2022_3008_MOESM1_ESM.docx]

**Additional file 1**

**Supplementary Materials:** Figure S1.


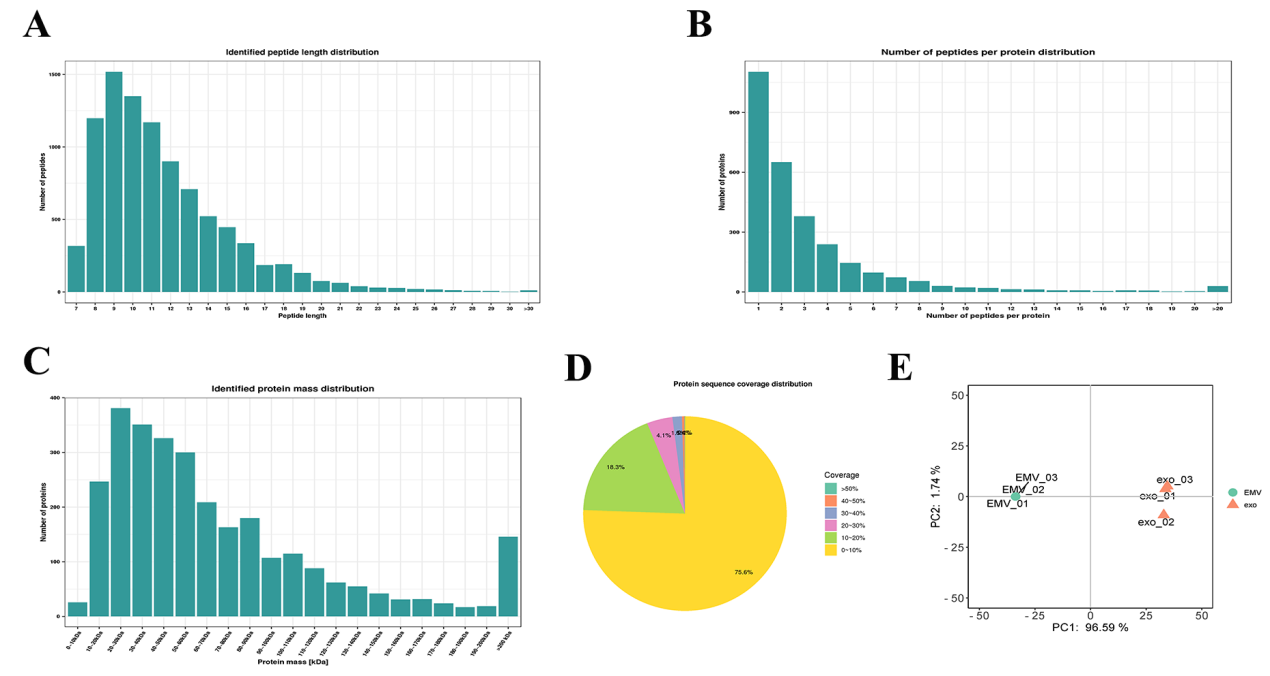


**Figure S1.** Sample repeatability test and quality control analysis of the mass spectrometry data. Peptide length distribution (A). Number of peptides per protein distributed (B). Mass distribution of the identified proteins (C). Protein sequence coverage distribution (D). PCA diagram (E).
